# Prognosis and recovery trajectories in prolonged disorders of consciousness: protocol for the PDOCC 5-year prospective cohort study

**DOI:** 10.3389/fneur.2026.1873014

**Published:** 2026-07-13

**Authors:** Hanbo Chen, Yong Luo, Weifeng Wen, Yunhong Deng, Zeyue Wen, Shujuan Huang, Li Qin, Si Chen, Xiao Lu

**Affiliations:** 1 Department of Rehabilitation Therapy, Guangdong Sanjiu Brain Hospital, Guangzhou, China; 2 Guangzhou Fengfan Information Technology Co., Ltd., Guangzhou, China; 3 Department of Rehabilitation Medicine, Guangdong Sanjiu Brain Hospital, Guangzhou, China

**Keywords:** 5-year, consciousness recovery, prolonged disorders of consciousness, prospective cohort study, protocol

## Abstract

**Background:**

Prolonged disorders of consciousness (pDoC), encompassing vegetative state (VS) and minimally conscious state (MCS), represent a formidable clinical challenge characterized by substantial uncertainty in prognostic assessment. Although prior studies have yielded several short-term observational findings, large-scale, long-term longitudinal data delineating the 5-year natural history of pDoC remain scarce—particularly within the Chinese healthcare context. Moreover, existing studies have frequently lacked standardized multimodal assessment protocols and have failed to account for competing risks (e.g., mortality) in statistical models, thereby limiting the accuracy of currently available prognostic tools. The present study aims to address this critical evidence gap through the establishment of a high-quality prospective cohort.

**Methods:**

The PDOCC study is a single-center, prospective cohort investigating 5-year disease trajectories in 1,000 patients (aged 18–80) with prolonged disorders of consciousness (VS or MCS ≥ 28 days post-brain injury) (traumatic and non-traumatic etiologies) enrolled at Guangdong Sanjiu Brain Hospital. Data collection includes standardized clinical assessments (CRS-R, GOS-E, DRS, EQ-5D, SF-36), neuroimaging (MRI/DTI/fMRI), and biomarkers (NfL, GFAP, S100B). Follow-up assessments at 3, 6, 12, 24, 36, 48, and 60 months combine in-person evaluations (all timepoints) with remote telephone interviews (3-month intervals after the 6-month visit). Neuroimaging and biomarkers are assessed at baseline and at 6, 12, 24, 36, 48 and 60 months. Primary outcomes are: (1) consciousness recovery, defined as emergence from MCS (eMCS) according to the CRS-R criteria (i.e., consistent functional communication and/or functional object use) sustained for ≥4 consecutive weeks; (2) all-cause mortality; and (3) major cardiovascular events (myocardial infarction, heart failure hospitalization, stroke). Secondary outcomes include: (1) functional status changes (GOS-E, DRS); (2) dynamic biomarker changes (NfL, S100B, GFAP); (3) health-related quality of life (EQ-5D); and (4) rehospitalization rate. Time-to-event analyses will use Fine-Gray competing-risk models to account for mortality as a competing event. Prognostic prediction modeling will be conducted in three sequential stages: a clinical model, a neuroimaging-augmented model (DTI/fMRI), and a fully integrated multimodal model additionally incorporating serum biomarkers (NfL, GFAP, S100B).

**Discussion:**

The PDOCC study will generate the first comprehensive longitudinal dataset characterizing long-term (5-year) outcomes of pDoC in a southern Chinese population. Through the incorporation of competing-risk analysis and a hybrid follow-up strategy, this protocol mitigates methodological biases that are prevalent in disorders of consciousness (DoC) research. The prognostic prediction models developed are anticipated to inform individualized rehabilitation decision-making, optimize the allocation of healthcare resources, and provide a methodological framework for future multicenter collaborative investigations.

**Clinical trial registration:**

https://www.chictr.org.cn/bin/project/edit?pid=246240, Identifier ChiCTR2400091315.

## Introduction

### Background and rationale

Prolonged disorders of consciousness (pDoC) constitute a clinical syndrome resulting from severe acquired brain injury, encompassing a diagnostic spectrum from vegetative state (VS) and minimally conscious state (MCS; further divided into MCS − and MCS+) through to emergence from MCS (eMCS) and, in the context of traumatic brain injury, post-traumatic confusional state (PTCS) as upper-boundary transitional states (1). Traumatic brain injury (TBI) represents one of the most prevalent etiologies of DoC, inducing diffuse axonal injury and multifocal cortico-subcortical network dysfunction that culminate in sustained impairment of conscious awareness (2). Although a subset of patients achieves meaningful functional recovery within the first year following injury, prolonged disorders of consciousness continue to pose a formidable clinical challenge. Notably, early functional deficits in the acute aftermath of severe brain injury do not necessarily portend an unfavorable long-term prognosis; for instance, among patients with moderate-to-severe TBI, the majority recover consciousness within 12 months even when an initial vegetative state is observed (3).

Multiple critical knowledge gaps persist in the current management of DoC. Neuroimaging and neurophysiological techniques have demonstrated capacity to detect “covert consciousness” in up to 15–20% of behaviorally unresponsive patients; however, routine clinical deployment of these modalities remains constrained by technical complexity, cost, and the requisite expertise for interpretation (1). Although functional neuroimaging modalities—including functional MRI (fMRI), electroencephalography (EEG), and positron emission tomography (PET)—have been shown to facilitate diagnosis and prognostication, and to predict recovery potential under specific circumstances, substantial practical barriers impede their widespread implementation (4). Furthermore, while multimodal assessment has been validated as a reliable predictor of unfavorable neurological outcomes in comatose survivors of cardiac arrest, the applicability of such strategies to DoC arising from alternative etiologies—such as stroke or hypoxic–ischemic encephalopathy—remains uncertain (5). Collectively, these limitations underscore the urgent need for high-quality prospective investigations that integrate standardized clinical assessment, advanced neurological monitoring, and longitudinal follow-up data.

### Study objectives

This study aims to systematically characterize the 5-year natural history of pDoC through a prospective cohort design, thereby bridging the critical evidence gap stemming from the absence of large-scale, long-term follow-up investigations. The specific objectives are as follows:

1. To determine the rates of consciousness recovery, all-cause mortality, and major cardiovascular events over a 5-year follow-up period in patients stratified by initial level of consciousness (VS, MCS−, and MCS+); primary comparisons will be conducted at the VS vs. MCS level, with MCS − vs. MCS + differentiation examined in prespecified subgroup analyses.
2. To identify etiology, injury severity (GCS at admission), time from injury to enrollment, Coma Recovery Scale–Revised (CRS-R) subscale profiles, and neuroimaging indices as independent predictors of consciousness recovery and unfavorable outcomes.
3. To develop and validate a multimodal prognostic prediction model integrating clinical, neuroimaging, and biomarker data.

The primary research question of this study is: among patients with prolonged disorders of consciousness (VS or MCS ≥ 28 days post-acute brain injury), does baseline consciousness level (VS vs. MCS) independently predict 5-year consciousness recovery trajectory, after adjustment for etiology, injury severity, and neuroimaging characteristics?

## Methods

### Study design and setting

The Prolonged Disorders of Consciousness Cohort (PDOCC) study is a single-center, prospective cohort study designed to systematically characterize the 5-year natural history and prognostic determinants of VS and MCS. A total of 1,000 eligible participants are planned for enrollment between June 2026 and May 2029, with longitudinal follow-up conducted over 5 years via structured telephone interviews at 3-month intervals. The study will be conducted at the Department of Rehabilitation Medicine, Guangdong Sanjiu Brain Hospital. The enrollment period spans from June 1, 2026 to May 31, 2029 (36 months). The follow-up period is calculated from each participant’s date of enrollment and extends for 60 months, with full cohort follow-up to be completed by May 31, 2034. Data collection encompasses a baseline assessment at enrollment and scheduled follow-up visits at 3, 6, 12, 24, 36, 48, and 60 months. The study protocol has been approved by the Institutional Review Board of Guangdong Sanjiu Brain Hospital (Ethics Approval No. 2026-01-073). Written informed consent will be obtained from all participants or their legally authorized representatives (LARs) prior to enrollment.

### Study population

The target population comprises individuals currently diagnosed with pDoC—specifically VS or MCS (encompassing both MCS − and MCS+)—at the time of enrollment. Patients who have already achieved eMCS prior to enrollment are not eligible, as eMCS constitutes the primary consciousness recovery endpoint of this study. Patients with post-traumatic confusional state (PTCS), which represents a transitional state beyond eMCS in the TBI recovery trajectory, are likewise not eligible for enrollment. Both conditions are characterized by persistent alterations in consciousness following severe acquired brain injury: VS is defined by wakefulness without awareness, whereas MCS indicates the preservation of partial conscious awareness.

Inclusion criteria are as follows: (1) a confirmed diagnosis of DoC with a duration of at least 28 days, established according to the 2020 European Academy of Neurology (EAN) guidelines (6) and assessed using the validated Chinese version of the CRS-R (7), including vegetative state (VS) and minimally conscious state (MCS; encompassing both MCS − and MCS+); (2) age between 18 and 80 years; (3) provision of written informed consent by the participant (if possessing decision-making capacity) or by a legally authorized representative (LAR); and (4) availability of complete medical records documenting the etiology of the initial brain injury, injury severity, and relevant diagnostic investigations. Eligible etiologies include all forms of acquired brain injury that can lead to prolonged disorders of consciousness (pDoC), such as traumatic brain injury (including both closed and penetrating head injuries) and non-traumatic brain injury—including hypoxic-anoxic injury (e.g., due to cardiac arrest, respiratory failure, or near-drowning), cerebrovascular events (such as ischemic stroke, hemorrhagic stroke, and subarachnoid hemorrhage), and metabolic or toxic encephalopathies.

Exclusion criteria include: (1) transient disorders of consciousness resulting from acute brain injury with a duration of fewer than 28 days; (2) severe organ dysfunction, defined as New York Heart Association (NYHA) Class III–IV heart failure, Child-Pugh Class C cirrhosis, or end-stage renal disease with an estimated glomerular filtration rate (eGFR) < 15 mL/min/1.73 m^2^; (3) irreversible end-stage disease, including TNM Stage IV metastatic malignancy or progressive neurodegenerative disease (e.g., Alzheimer’s disease, Parkinson’s disease, amyotrophic lateral sclerosis), given that consciousness impairment in these conditions may reflect primary disease progression rather than post-injury pDoC; (4) inability to comply with the study protocol, such as residing more than 200 km from the study site, presence of severe communication barriers, or explicit refusal of follow-up; and (5) severe comorbid orthopedic or internal injuries (e.g., unstable spinal fracture, severe polytrauma with active hemorrhagic shock) that preclude safe administration of CRS-R or neuroimaging protocols, or that are judged by the attending neurologist to render behavioral consciousness assessment invalid. Patients with non-confounding orthopedic injuries (e.g., healed limb fractures not affecting motor command assessment) are not excluded; all such comorbidities will be documented and incorporated as covariates. Pre-existing stable neurological diagnoses (e.g., prior cerebrovascular events, epilepsy) will be recorded as comorbidities and adjusted for in all multivariable models rather than treated as grounds for exclusion.

### Recruitment procedures

All participants will be recruited from the Department of Rehabilitation Medicine, Guangdong Sanjiu Brain Hospital. The recruitment process follows a three-step procedure: first, potential participants will be identified through referrals from neurologists, rehabilitation specialists, and the hospital’s internal referral network, with eligibility screening based on medical record review and preliminary clinical assessment; second, eligible candidates will be contacted by a research coordinator, who will provide their legally authorized representatives with a detailed explanation of the study objectives, procedures, risks, and benefits, and written informed consent will be obtained; finally, enrolled participants will undergo a comprehensive baseline assessment—including demographic characteristics, detailed medical history, current clinical status (CRS-R score), neuroimaging investigations, and laboratory testing—conducted collaboratively by the research team comprising neurologists, rehabilitation therapists, and research nurses.

### Variables

#### Primary and secondary outcome variables

The primary outcome variables of this study are as follows: (1) pDoC recovery, defined as emergence from MCS (eMCS), operationalized as achieving a CRS-R communication subscale score ≥2 (functional interactive communication) or a motor subscale score ≥6 (functional object use), confirmed on ≥2 standardized CRS-R assessments conducted ≥7 days apart and sustained for ≥4 consecutive weeks. This criterion applies uniformly to patients with a baseline diagnosis of VS or MCS. All recovery determinations will be adjudicated by the independent Endpoint Adjudication Committee; (2) all-cause mortality, defined as death from any cause at any point during the 5-year follow-up period (up to and including month 60). In the competing-risk analytical framework, all-cause mortality serves as the primary competing risk event for the consciousness recovery outcome. Patients who survive to the end of the 60-month follow-up without having achieved the consciousness recovery endpoint will be administratively right-censored at month 60; and (3) major cardiovascular events (MACE), encompassing myocardial infarction (defined in accordance with the Fourth Universal Definition of Myocardial Infarction, requiring elevated cardiac biomarkers in conjunction with clinical evidence of myocardial ischemia), hospitalization for heart failure (defined as inpatient admission requiring intravenous diuretics or inotropic support), and stroke (confirmed by neuroimaging [CT/MRI] with neurological deficits persisting for more than 24 h).

Secondary outcome variables include: (1) changes in functional status, assessed using the Glasgow Outcome Scale–Extended (GOS-E) and the Disability Rating Scale (DRS) (8, 9); (2) dynamic changes in biomarkers, including serum neurofilament light chain (NfL), glial fibrillary acidic protein (GFAP), S100 calcium-binding protein B (S100B), and other injury- and recovery-related biomarkers; (3) health-related quality of life, evaluated using the EQ-5D questionnaire, with caregiver proxy responses employed when direct patient assessment is not feasible; and (4) rehospitalization rate, documented by recording the frequency and causes of readmission, with particular attention to common complications such as infections, seizures, and respiratory events.

#### Exposure variable

The primary exposure variable is the baseline level of consciousness (VS or MCS) at enrollment. All enrolled participants will receive standardized multimodal rehabilitation care in accordance with the Chinese Expert Consensus on the Diagnosis and Treatment of Prolonged Disorders of Consciousness (Standard of Care, SOC) (10), encompassing: (1) pharmacological arousal-promoting therapy (e.g., amantadine, donepezil, zolpidem); (2) multimodal sensory stimulation therapy (auditory, tactile, visual, and olfactory modalities); (3) physical therapy (including tilt-table training and passive mobilization); (4) occupational therapy; (5) speech-language therapy; (6) hyperbaric oxygen therapy (where indicated and available); and (7) neuromodulation techniques (e.g., median nerve electrical stimulation, repetitive transcranial magnetic stimulation, spinal cord stimulation), as well as evidence-based complication management (infections, spasticity, pressure injury). Treatment intensity for each modality will be quantified as total weekly contact time (minutes per week) and session frequency, recorded prospectively in the electronic data capture system at each assessment timepoint. All treatment variables will be incorporated as time-dependent covariates (operationalized as weekly contact time (min/week) per rehabilitation modality) in the analytical models to distinguish the natural disease course from treatment-mediated effects.

#### Predictor variables and covariates

Primary prognostic predictor variables have been selected based on established clinical and biological plausibility for predicting consciousness recovery: (1) etiology of brain injury (TBI, hypoxic-ischaemic encephalopathy, cerebrovascular event, metabolic/toxic encephalopathy, other), given that neurobiological recovery trajectories differ substantially across aetiological subgroups; (2) injury severity at the acute phase (GCS score at hospital admission); (3) time from acute brain injury to study enrollment; (4) CRS-R subscale scores at baseline (auditory, visual, motor, oromotor/verbal, communication, and arousal subscales); and (5) neuroimaging characteristics (white matter integrity on DTI, functional connectivity on fMRI). Pre-injury lifestyle characteristics [smoking status, alcohol consumption, physical activity (assessed via caregiver proxy report using IPAQ) (11), and dietary habits] are retained as exploratory secondary covariates; their collection is hypothesis-generating and their role as determinants of post-injury recovery will be interpreted with appropriate caution given the reliance on caregiver proxy report.

Pre-specified adjustment covariates in all multivariable models include age, sex, educational level, and comorbidities (e.g., cardiovascular disease, diabetes mellitus, chronic kidney disease). It should be noted that injury severity (GCS at acute admission) and the interval from injury to enrollment are designated as primary prognostic predictor variables in this study (see above), rather than as confounders, given their established direct effects on consciousness recovery trajectories; they will therefore enter the analytical models as predictors of interest rather than as adjustment terms. Effect modifiers—including age stratum (< 50 years vs. ≥50 years), injury type (traumatic vs. non-traumatic), and baseline CRS-R score—will be formally evaluated through prespecified subgroup analyses and interaction testing.

### Data sources and measurements

All baseline data will be collected within 2 weeks of enrollment at the Department of Rehabilitation Medicine, Guangdong Sanjiu Brain Hospital, to ensure data consistency and minimize recall bias.

Demographic, lifestyle, medical history, and family history data will be collected via structured questionnaires administered by trained research personnel during face-to-face interviews. Questionnaire items include: age (years), sex (male/female), educational attainment (primary/secondary/tertiary), occupation (employed/retired/unemployed), marital status (single/married/divorced/widowed), smoking status (never/former/current), alcohol consumption (frequency and quantity), physical activity level [assessed by IPAQ (11)], dietary habits (frequency of fruit, vegetable, and processed food intake), detailed prior diagnoses (TBI, stroke, hypoxic–ischemic encephalopathy, etc.), current medication use (drug name, dose, and duration), and relevant disease history in first-degree relatives.

Physical examinations will be performed under standardized conditions by a consistent team of trained physicians to minimize measurement variability. Anthropometric measurements include height (measured to ±0.1 cm using a stadiometer), body weight (measured to ±0.1 kg using a calibrated electronic scale), body mass index (BMI, calculated as weight [kg]/height [m]^2^), and waist and hip circumferences (measured with a flexible tape). Blood pressure will be measured using an automated sphygmomanometer after at least 5 min of rest, with the mean of two to three readings recorded at 1-min intervals. Additional vital signs include heart rate (beats per minute) and body temperature (°C).

Laboratory analyses will be performed on blood and urine samples collected following overnight fasting, processed according to standardized protocols at the accredited clinical laboratory of Guangdong Sanjiu Brain Hospital. Blood tests include: complete blood count (CBC); lipid profile (total cholesterol, low-density lipoprotein cholesterol [LDL-C], high-density lipoprotein cholesterol [HDL-C], and triglycerides); fasting plasma glucose; glycated hemoglobin (HbA1c); renal function (estimated glomerular filtration rate [eGFR], calculated using the CKD-EPI equation); and disease-specific biomarkers, including inflammatory markers (C-reactive protein [CRP] and interleukin-6 [IL-6]) and neurotrophic factors (brain-derived neurotrophic factor [BDNF]). Urinalysis includes routine urinalysis and urine albumin-to-creatinine ratio (ACR) to evaluate renal function and detect early signs of complications.

### Neuroimaging and neurophysiological assessment protocols

Structural MRI (3 T): T1-weighted (MPRAGE, 1 mm isotropic), T2-weighted FLAIR, and susceptibility-weighted imaging (SWI) will be acquired using a standardized protocol on a 3 T Siemens Prisma scanner. Lesion localization and volumetrics will be quantified using FreeSurfer v7.4 (cortical thickness, subcortical volumes) and SPM12 (lesion overlay analysis).

Diffusion Tensor Imaging (DTI): 64-direction DTI will be acquired (b = 1,000 s/mm^2^) and preprocessed using FSL v6.0 (eddy current correction, motion correction, tensor fitting). Primary indices: fractional anisotropy (FA) and mean diffusivity (MD) in the corticospinal tract, thalamocortical projections, and corpus callosum, derived using tractography with the MNI white matter atlas as reference.

Resting-State fMRI (rs-fMRI): A 10-min eyes-closed resting-state acquisition (TR = 2 s, TE = 30 ms, 3 mm isotropic) will be preprocessed using fMRIPrep v23.2 (motion scrubbing: FD > 0.5 mm, DVARS > 1.5; band-pass filtering: 0.01–0.1 Hz; spatial smoothing: 6 mm FWHM). Primary derived indices: (1) default mode network (DMN) internal functional connectivity (seed-based analysis using posterior cingulate cortex as reference seed); (2) thalamocortical connectivity (bilateral thalamic seeds); and (3) amplitude of low-frequency fluctuations (ALFF).

EEG: 64-channel EEG will be acquired during a 30-min resting-state paradigm and preprocessed using EEGLAB v2023.0 (independent component analysis for artifact rejection; re-referencing to average; band-pass filtering: 0.5–40 Hz). Primary derived indices: (1) spectral power in delta (0.5–4 Hz), theta (4–8 Hz), alpha (8–13 Hz), and beta (13–30 Hz) bands; (2) microstate analysis (4-class canonical microstates, mean duration, frequency, and global explained variance); and (3) perturbational complexity index (PCI).

Covert Consciousness Detection Protocol: Motor imagery (MI) paradigms will be administered to all participants with sufficient behavioral arousal (CRS-R arousal subscore ≥1). The standardized paradigm requires participants to imagine squeezing their right hand (“yes”) or wiggling their toes (“no”/rest) in response to binary yes/no questions. EEG data will be analyzed using a support vector machine (SVM) classifier with 10-fold cross-validation (accuracy threshold: ≥ 70% for positive classification). fMRI motor imagery (supplementary motor area [SMA] activation) will serve as concurrent validation. All paradigms will be administered and scored by two independently trained operators blind to each other’s results; inter-rater reliability will be quantified using Cohen’s kappa.

### Follow-up procedures

Follow-up assessments will employ a hybrid surveillance strategy combining in-person evaluations with remote monitoring. Core neurobehavioral and functional assessments (CRS-R, GOS-E, DRS, EQ-5D, SF-36, FIM, HADS, and Zarit Caregiver Burden Interview) will be conducted at bedside by certified assessors at baseline and at 3, 6, 12, 24, 36, 48, and 60 months (8 assessment timepoints in total, comprising 7 in-person follow-up visits). The Chinese validated CRS-R (7) will be used throughout. Assessors must complete standardized training—covering scoring, ≥10 supervised assessments, and ICC ≥ 0.85 reliability—before independent use. The same assessor will evaluate each participant at all timepoints when feasible; if replaced, inter-rater reliability will be verified against the prior assessor’s last two evaluations. Total and subscale scores will be recorded at each visit. eMCS is defined by subscale-specific behavioral thresholds (per Primary Outcome Variables), not a total score of 23/23, which reflects full neurological recovery rather than functional communication or object use. Neuroimaging investigations (MRI/DTI/fMRI) and serum biomarkers (NfL, S100B, GFAP, and other injury-related biomarkers) will be performed at baseline and at 6, 12, 24, 36, 48 and 60 months. Following the 6-month visit, remote follow-ups will be conducted by telephone interview supplemented by caregiver-reported outcomes at 3-month intervals between scheduled in-person visits, primarily capturing vital status, rehospitalization events, and major adverse events. Remote follow-ups will be administered by uniformly trained research coordinators using standardized scripts, with video calls employed as an adjunct to confirm the patient’s general condition. In-person outpatient visits will be conducted by neurologists and rehabilitation therapists.

Follow-up assessments will capture: changes in consciousness level (CRS-R); vital status; major cardiovascular events; functional independence (Functional Independence Measure [FIM]); health-related quality of life (SF-36); caregiver burden (Zarit Caregiver Burden Interview); changes in medication or treatment regimens; activities of daily living (ADLs) and instrumental activities of daily living (IADLs); mental health status (Hospital Anxiety and Depression Scale [HADS]); and adverse event documentation. All follow-up data will be entered in real time into an electronic data capture (EDC) system, with monthly data quality reviews conducted to achieve a target follow-up completion rate of ≥ 85%.

Outcome event ascertainment procedures are as follows: recovery status will be confirmed through a composite of clinical assessments, medical record review, and neuroimaging evidence (e.g., fMRI or EEG demonstrating improved cortical activity); death events will be ascertained via civil death registration systems, family-reported notification, and hospital records, with cause-specific mortality classified as neurological deterioration, cardiovascular events, infection, or other causes; cardiovascular events will be adjudicated according to internationally accepted definitions supported by neuroimaging and laboratory evidence. Telephone interviews have been established as a feasible follow-up modality, demonstrating agreement with self-reported outcome measures in the majority of settings, achieving a cohort retention rate of 93% within 6 months in culturally adapted intervention research, and proving applicable to the longitudinal assessment of functional and cognitive recovery trajectories following neurological infections (12) (Table 1).

**Table 1:** Evaluation parameters at baseline and follow-up visits.

Evaluation parameter	Baseline (enrollment)	Follow-up (telephone/video or on-site visits)						
	(Hospital admission)	Visit 1 (3 m)	Visit 2 (6 m)	Visit 3 (12 m)	Visit 4 (24 m)	Visit 5 (36 m)	Visit 6 (48 m)	Visit 7 (60 m)
Informed consent	x							
Inclusion criteria met	x							
Demographics & medical history	x							
Vital signs & neurological exam	x							
Consciousness level (CRS-R)	x	x	x	x	x	x	x	x
Functional status (GOS-E, DRS)	x	x	x	x	x	x	x	x
Quality of life (EQ-5D)	x	x	x	x	x	x	x	x
Survival status	x	x	x	x	x	x	x	x
Rehospitalization & complications	x	x	x	x	x	x	x	x
Adverse events	x	x	x	x	x	x	x	x
Treatment records (SOC, medications, neuromodulation, including weekly contact time per discipline)	x	x	x	x	x	x	x	x
Neuroimaging (MRI/DTI/fMRI)	x			x	x	x	x	x
Biomarkers (NfL, S100B, GFAP)	x			x	x	x	x	x
Laboratory tests (CBC, electrolytes, etc.)	x							
Primary outcomes (consciousness recovery, all-cause death, major CV events)		x	x	x	x	x	x	x

× Represents data collection timepoint. CRS-R, Coma Recovery Scale-Revised; GOS-E, Extended Glasgow Outcome Scale; DRS, Disability Rating Scale; EQ-5D, EuroQol 5-Dimensions; SOC, Standard of Care; NfL, Neurofilament Light Chain; GFAP, Glial Fibrillary Acidic Protein; CBC, Complete Blood Count.

### Bias control

To minimize selection bias, a systematic three-step recruitment procedure (screening, informed consent, and baseline assessment) will be implemented with independent review by a multidisciplinary team. To address follow-up bias, a hybrid surveillance strategy combining telephone interviews and outpatient visits will be employed, with a target follow-up completion rate of ≥ 85% and active tracing of participants lost to follow-up. Confounding bias will be controlled through multivariable regression models incorporating documented potential confounders, with effect modification explored in prespecified subgroup analyses. To mitigate performance and detection bias, outcome assessors will be blinded to baseline results; all primary outcome events will be adjudicated by an independent Endpoint Adjudication Committee in a blinded manner; and all neuroimaging data will be independently reviewed by two radiologists. The study has been prospectively registered on an international clinical trial registry and will be reported in accordance with the STROBE statement.

### Sample size calculation

The sample size was determined based on 5-year consciousness recovery rates reported in the existing literature on prolonged disorders of consciousness. Chen et al. (13) reported a 5-year consciousness recovery rate of 42.3% in a prospective cohort of Chinese patients; a systematic review by Giacino et al. (14) indicated recovery rates of approximately 35–50% among patients with pDoC following TBI; and a recent longitudinal study by Yan et al. (15) reported a 5-year cumulative recovery rate of 48.7%. Given that the present study enrolls a mixed-etiology cohort (traumatic and non-traumatic) and employs a competing-risk analytical framework, a conservative 5-year cumulative incidence rate of 34% (annual event rate: 8%) was adopted as the basis for sample size estimation, ensuring adequate statistical power. Assuming a hazard ratio (HR) of 1.3, two-sided *α* = 0.05, 80% statistical power, and an exposure proportion of 30%, the required number of events was calculated as 544 using the Schoenfeld formula. Adjusting for an anticipated 15% loss to follow-up over the 5-year period, the target sample size was determined to be 1,000 participants. This sample size will ensure adequate statistical power for the primary survival outcome, support detection of secondary outcomes (functional recovery and quality-of-life changes), enable the identification of moderate effect sizes (Cohen’s *d* ≥ 0.5 or OR ≥ 1.5), and accommodate prespecified subgroup analyses. Prespecified subgroup analyses will evaluate the influence of age, sex, and baseline severity; assuming a subgroup prevalence of 30% and an event rate consistent with the overall cohort, ≥ 80% power will be achievable for detecting HR ≥ 1.5 within each subgroup.

## Statistical analysis methods

### General principles and descriptive statistics

All statistical analyses will employ two-sided tests with a significance threshold of α = 0.05, and results will be reported with 95% confidence intervals. Bonferroni correction or false discovery rate (FDR) adjustment will be applied to control Type I error inflation in the context of multiple comparisons. Multivariable regression models will be constructed within the competing-risk framework to identify independent prognostic predictors of consciousness recovery, as detailed in the Primary Analysis subsection below. Separately, a dedicated multimodal prognostic prediction model will be developed and validated using a three-stage hierarchically nested approach incorporating LASSO-based variable selection and bootstrap internal validation (1,000 resampling iterations); the C-index will serve as the primary discrimination metric. The full construction procedure for this prediction model is described in the dedicated subsection below.

### Primary analysis

For the primary outcome of consciousness recovery, a competing-risk analytical framework will be applied, in which consciousness recovery is designated as the event of interest and all-cause mortality as the competing risk event. Cumulative Incidence Functions (CIFs) will be estimated, and the Fine-Gray subdistribution hazard model will be used to assess the influence of each predictor on the subdistribution hazard of recovery. For the single-endpoint outcome of all-cause mortality, Cox proportional hazards regression will be employed. Covariates will be introduced in a stepwise fashion across five models: Model 1 (unadjusted); Model 2 (adjusted for demographic variables: age and sex); Model 3 (Model 2 plus clinically relevant injury-related variables: etiology [TBI vs. HIE vs. cerebrovascular vs. metabolic/toxic encephalopathy vs. other], GCS at acute admission, and time from injury to enrollment); Model 4 (Model 3 plus baseline CRS-R total score and neuroimaging indices [DTI-FA and fMRI-DMN connectivity]); and Model 5 (fully adjusted model incorporating all prespecified covariates: age, sex, educational level, comorbidities, etiology of brain injury, GCS at acute admission, time from injury to enrollment, baseline CRS-R total score, and neuroimaging indices). Covariate selection will be guided by directed acyclic graph (DAG) construction and prior literature evidence to avoid bias introduced by over-adjustment of mediators and colliders.

The proportional hazards assumption will be tested using Schoenfeld residuals and log–log survival plots; violations will be addressed through stratified Cox models, time-varying covariates, or restricted cubic splines. Results will be expressed as hazard ratios with 95% confidence intervals. Kaplan–Meier curves will be used to visualize survival probabilities across exposure groups, and forest plots will be employed to display effect estimates and their trends across adjusted models.

In addition to the primary time-to-event analysis, a pre-specified nested prognostic comparison will be conducted between participants who achieve the consciousness recovery endpoint and those who do not (either remaining in VS/MCS or experiencing death as a competing event), using the Fine-Gray subdistribution hazard model incorporating all primary prognostic predictor variables (etiology, GCS at acute admission, time from injury to enrollment, CRS-R subscale scores at baseline, and neuroimaging indices). This nested comparison is designed to identify the multivariate prognostic profile that discriminates recovery-positive from recovery-negative trajectories within the cohort.

### Multimodal prognostic prediction model construction

A multimodal prognostic prediction model will be constructed in three hierarchically nested stages: (1) a Clinical Model incorporating demographic and injury-related variables (age, sex, etiology, GCS at admission, time from injury to enrollment, and baseline CRS-R total score) selected via LASSO regression with 10-fold cross-validation; (2) a Neuroimaging-Augmented Model adding DTI-derived fractional anisotropy (corticospinal tract and corpus callosum) and resting-state fMRI default mode network connectivity to Stage 1; and (3) a Fully Integrated Multimodal Model further incorporating baseline serum biomarkers (NfL, GFAP, S100B). Model discrimination will be quantified by the C-index; incremental value across stages will be assessed using IDI and NRI (95% CIs via bootstrap resampling, 1,000 iterations). The final model will undergo bootstrap internal validation with optimism correction, and calibration will be evaluated using the Harrell calibration plot.

### Secondary analyses and longitudinal data analysis

Secondary outcomes will be analyzed using methods appropriate to each variable type: changes in functional status scores will be modeled using linear mixed-effects models or generalized estimating equations (GEE) to account for within-individual correlations in repeated-measures data; dynamic biomarker changes will be assessed using repeated-measures analysis of variance (ANOVA); quality-of-life scores will be analyzed using generalized linear models; and rehospitalization rates will be modeled using negative binomial regression. All models will account for time effects and between-individual variability.

### Subgroup and interaction analyses

Prespecified subgroup analyses will be conducted based on clinical relevance and potential effect modification, including: age (< 50 years vs. ≥ 50 years), sex (male vs. female), injury type (traumatic vs. non-traumatic), and baseline level of consciousness (VS vs. MCS). Interaction terms (product terms between the exposure variable and subgroup variables) will be introduced into multivariable regression models to formally test the statistical significance of interactions, with a significance threshold of *p* < 0.10. Statistically significant interactions will be further examined through stratified subgroup analyses, with results displayed as forest plots showing HRs and 95% CIs for each subgroup. All subgroup analyses will be characterized as exploratory findings to prevent overinterpretation.

### Missing data handling

The distribution and potential mechanisms of missing data will be systematically evaluated, with reporting of the proportion of missingness per variable, the missing data pattern (monotone or non-monotone), and comparisons of baseline characteristics between participants with and without missing data. Primary analyses will assume data missing at random (MAR): complete case analysis will be applied when the proportion of missing data is below 5%; when missingness exceeds 5%, multiple imputation by chained equations (MICE) will be performed using 20 imputation datasets, with imputation models incorporating all relevant variables, and results pooled using Rubin’s rules. Sensitivity analyses will employ pattern mixture models (with *δ*-adjustment parameters) and tipping point analyses to evaluate the robustness of study conclusions under alternative missing data assumptions.

### Loss to follow-up

A standardized definition of loss to follow-up will be applied (three consecutive missed follow-up contacts). Data from participants lost to follow-up will be right-censored at the time of their last valid contact. Reasons for loss to follow-up (relocation, withdrawal, death, loss of contact, etc.) will be systematically documented. Baseline demographic and clinical characteristics will be compared between participants lost to follow-up and those who complete follow-up to assess potential selection bias. Inverse probability weighting (IPW) will be applied as needed to statistically correct for informative censoring due to dropout, thereby enhancing the external validity and generalizability of study findings.

### Sensitivity analyses

All sensitivity analyses are prespecified in the study protocol to prevent post-hoc analytical bias. The analytical strategies include: (1) application of alternative outcome definitions (e.g., modified recovery criteria or endpoint adjudication standards); (2) analysis of alternative study populations (per-protocol analysis and as-treated analysis); (3) application of the cause-specific hazard model as an alternative to the Fine-Gray subdistribution hazard model for the primary competing-risk analysis; (4) comparison of results across different missing data handling approaches (complete case analysis, multiple imputation, and single imputation); and (5) evaluation of the impact of alternative covariate adjustment strategies (stepwise adjustment, full model, and reduced model) on effect estimates. Through systematic comparison of results across varying assumptions and analytical methods, the robustness and reliability of study conclusions will be comprehensively assessed.

## Discussion

The field of pDoC currently faces two fundamental challenges: first, the absence of large-scale, long-term longitudinal follow-up data in Chinese populations—as existing evidence is predominantly derived from European and North American cohorts (16, 17) and therefore inadequately reflects the disease trajectories shaped by China’s distinct etiological profile, healthcare resource distribution, and rehabilitation culture; second, the predominance of prognostic prediction models based on cross-sectional or short-term follow-up data, which are unable to capture the dynamic evolution of consciousness recovery and consequently deprive clinical decision-making of accurate temporal guidance, resulting in considerable uncertainty in the timing of therapeutic interventions (18). To address these challenges, we designed this single-center, prospective, observational cohort study with a 5-year follow-up period, anchored at the Department of Rehabilitation Medicine, Guangdong Sanjiu Brain Hospital—a nationally recognized leading center for pDoC diagnosis and management. The central objective of this protocol is to establish a high-quality, longitudinal database of long-term outcomes in Chinese patients with pDoC, enabling the systematic characterization of dynamic changes in consciousness recovery, quality of life, and functional status, and the construction of precision prognostic prediction models tailored to the domestic population, thereby providing an evidence base for individualized intervention strategies.

This study achieves several important methodological advances. The rigorous prospective longitudinal design, with a 5-year follow-up period substantially exceeding that of most published studies (typically 1–2 years), enables comprehensive characterization of both the critical time windows for consciousness recovery and the features of long-term stable plateaus—directly addressing the predominant reliance on cross-sectional observations in the field and providing unprecedented longitudinal data to elucidate the time-dependent nature of recovery. In terms of data integration, this protocol transcends the limitations of traditional single-source clinical data by harmonizing structured electronic medical records, standardized neuropsychological assessments (CRS-R), objective neuroimaging (structural and functional MRI), serum biomarkers of neural injury, and patient-reported outcomes (health-related quality of life instruments). Notably, the application of internationally standardized instruments with blinded assessment for the definition of core outcome variables substantially reduces misclassification and information bias. This multisource data integration is anticipated to reveal deep associations between clinical phenotypes and biological mechanisms, uncovering interaction patterns that conventional single-modality research cannot identify. To mitigate the most critical methodological threats in DoC research—attrition bias and measurement bias—we have implemented distinctive safeguards: a hybrid follow-up strategy combining telephone interviews and outpatient visits, with a quality target of ≥ 85% follow-up completion rate; uniform training and certification of all assessors; blinded adjudication of all primary outcome events by an independent Endpoint Adjudication Committee; and independent dual-reader review of all neuroimaging data. As a single-center study, we are able to achieve a highly standardized operational workflow and quality control system, with all data collection adhering to unified standard operating procedures (SOPs). The study has been prospectively registered on an international clinical trial registry and will be reported in compliance with the STROBE statement; the level of quality control substantially exceeds the average for comparable observational studies.

The choice of a prospective cohort design was deliberate and methodologically grounded. Prospective cohort design is the recognized standard for pDoC natural history and prognostic investigation, as reflected by all landmark studies cited in this protocol (13–17). More fundamentally, the Fine-Gray competing-risk model—a core analytical feature of this protocol—requires prospectively ascertained event-time data and is methodologically incompatible with a retrospective case–control framework. The prospective design additionally enables modeling of time-dependent covariates (notably, treatment intensity) to distinguish natural recovery trajectories from treatment-mediated change, and uniquely permits characterization of the temporal dynamics of recovery—information that is inaccessible to case–control designs. Nevertheless, to address the analytical goal of comparing patients who recover with those who do not, a pre-specified nested comparison between recovery-positive and recovery-negative groups will be conducted within the cohort framework using the Fine-Gray model with pre-specified clinical prognostic predictors.

The 5-year follow-up horizon was selected to align with the most comprehensive existing prospective pDoC cohorts (13, 15) reporting 5-year cumulative recovery rates of 42.3–48.7% in Chinese populations, and to capture late recovery events beyond the conventional 12-month observation window that dominates prior literature.

Despite these methodological strengths, several limitations warrant acknowledgment. As a single-center study, this protocol may face limitations in sample representativeness, and caution will be required when generalizing findings to other regions of China. Additionally, extended follow-up duration may increase attrition rates, and certain self-reported variables may be susceptible to recall bias. Detailed contingency measures have been developed to address these risks: an additional 20% recruitment buffer has been incorporated into the sample size calculation to accommodate anticipated dropout; multiple imputation by chained equations will be employed to handle missing data, and robustness will be evaluated using pattern mixture models and tipping point analyses; baseline characteristics will be compared between participants lost to follow-up and completers, with inverse probability weighting applied to correct for potential selection bias; objective medical records will be prioritized over self-reported data wherever feasible; and sensitivity analyses will be performed to validate the reliability of primary conclusions. The limitations of the single-center design will be explicitly acknowledged in the discussion, and future multicenter validation research will be recommended.

Upon successful completion of this protocol, the resulting data will provide critically needed, locally contextualized evidence to support pDoC diagnosis and management in Guangdong and the broader South China region. The study will supply high-quality parameters for consciousness recovery prediction models and promote a paradigm shift from empirical intervention toward precision timing of therapeutic strategies. As a leading single-center pDoC cohort in China, the standardized data collection workflow and quality control system established by this study will serve as a reference template for similar investigations at other institutions, facilitating the methodological standardization of pDoC research in China. Most importantly, the prognostic prediction tools developed from this study will provide individualized decision support for clinicians, assist patient families in planning rehabilitation pathways and resource allocation, and ultimately improve patient quality of life while reducing the societal burden of caregiving. This protocol establishes a methodological benchmark for high-quality single-center cohort research in the pDoC field and lays a robust data foundation and technical infrastructure for future multicenter collaborative investigations.
